# Theoretical Study on the Grafting Reaction of Benzophenone Compounds to Polyethylene in the UV Radiation Cross-Linking Process

**DOI:** 10.3390/polym17192595

**Published:** 2025-09-25

**Authors:** Yang Du, Chi Deng, Hui Zhang, Xia Du, Yan Shang, Xuan Wang

**Affiliations:** Key Laboratory of Engineering Dielectrics and Its Application of Ministry of Education, School of Material Science and Chemical Engineering, Harbin University of Science and Technology, Harbin 150080, China; duyang950711@163.com (Y.D.); dengchi@potevio.com (C.D.); duxia62@126.com (X.D.); shangyan1972@126.com (Y.S.); wangxuan@hrbust.edu.cn (X.W.)

**Keywords:** cross-linking polyethylene, grafting reaction, benzophenone compounds, density functional theory, transition state

## Abstract

In this study, benzophenone compounds substituted with electron-withdrawing groups (-NO_2_, -F, and -Cl) and electron-donating groups (-OH, -CH_3_, -NH_2_, and -OCH_3_) were employed as voltage stabilizers for crosslinked polyethylene (XLPE) insulation materials. At B3LYP/6-311+G(*d*,*p*) level, reaction Gibbs free potential energy data for eleven reaction channels and molecular characteristics, including electron affinity EA(s), ionization potential IP(s), and HOMO-LUMO gap (*E*_g_) of benzophenone derivatives, were obtained. The effects of electron-donating and electron-withdrawing functional groups were systematically evaluated. The calculated results indicate that benzophenones exhibit the lowest Gibbs free energy barrier for grafting onto polyethylene among the investigated molecules. With the introduction of electron-donating groups, the reaction Gibbs free energy barrier increases. It is worth noting that 2-Nitrobenzophenone is considered to possess superior electrical resistivity, attributed to its highest electron affinity among the studied compounds. This investigation is expected to provide reliable insights for the development of modified polyethylene-based insulating materials for high-voltage cables.

## 1. Introduction

Currently, the power grid is undergoing rapid development. Notably, renewable energy sources, such as solar and wind energy, are increasingly emerging as primary energy sources, which necessitates reliance on high-capacity long-distance power transmission technologies. Polymer-insulated cables exhibit advantages including excellent insulation performance, light weight, low cost, and good flexibility, thus gradually replacing oil-filled cables and mass-impregnated cables [1,2]. Crosslinked polyethylene (XLPE) is widely recognized as a common insulating material for high-voltage power cables. Owing to its superior electrical performance, it exhibits promising applications in high- and ultra-high-voltage fields [3]. Electrical treeing is one of the most critical factors causing performance degradation in cross-linked polyethylene (XLPE) cables [4,5,6,7]. In earlier studies, to further enhance the voltage rating of cross-linked polyethylene (XLPE) insulated power cables, conventional approaches included increasing insulation thickness, applying voltage stabilizers, or reducing the size and quantity of additives and the number of defects in XLPE insulation [2,8,9]. Super-clean technology has been successfully applied in polyethylene insulation for commercial products [10]. However, the super-clean method has approached its performance limit in polyethylene insulation applications. Over the past few decades, the incorporation of voltage stabilizers has been shown to improve the electrical tree resistance of XLPE insulation under high electric fields [8,11]. Voltage stabilizers exhibit dual functionality under high electric fields: they effectively dissipate the energy of high-energy electrons (hot electrons) through non-radiative relaxation processes and inhibit hot electrons from bombarding polyethylene molecular chains, thereby significantly elevating the initial breakdown voltage of XLPE insulation systems [12]. In comparison to conventional peroxide cross-linking processes, ultra-violet (UV)-irradiated polyethylene cross-linking technology presents distinct technical merits, including rapid processing kinetics (with cross-linking times reduced from hours to minutes), localized irradiation zones that minimize thermal damage to surrounding materials, and superior energy efficiency stemming from direct photon-driven reactions [13]. Furthermore, our previous theoretical investigations utilizing density functional theory (DFT) quantitatively validated the superiority of UV-irradiated cross-linking over traditional peroxide-initiated systems: the calculated reaction potential energy barrier for UV-induced cross-linking was 0.17 eV, which is 0.08 eV lower than that of peroxide-mediated cross-linking, indicating more favorable thermodynamic feasibility [14,15].

Voltage stabilizers typically belong to organic aromatic compounds, which mainly include polycyclic aromatic compounds and those featuring benzophenone-like structural motifs [11,16,17,18,19]. Among these, specific types have demonstrated remarkable efficacy in enhancing insulation performance: for example, an optimal dosage of acetophenone-based voltage stabilizer has been shown to significantly improve the alternating current (AC) breakdown strength of cross-linked polyethylene (XLPE) insulation by up to 50% [20]. The molecular design of voltage stabilizers is guided by fundamental electronic properties. As reported in existing studies, high electron affinity has been established as a key guiding principle for developing efficient voltage stabilizers [12,21,22]. This principle is further validated by comparative analysis of representative compounds: benzophenone (Bp), for instance, exhibits an electron affinity approximately 0.40 eV higher than that of acetophenone, underscoring its potential superiority in electron energy dissipation under high electric fields [15,20].

2-Hydroxybenzophenone derivatives are key ultra-violet absorbers (UVAs) widely used for photostabilizing traditional polymers, like plastics and coatings. Derived primarily from 2,4-dihydroxybenzophenone, they require retaining the *ortho*-hydroxyl group adjacent to the carbonyl moiety to keep efficient photostabilization. This group forms an intramolecular hydrogen bond, facilitating excited-state intramolecular proton transfer (ESIPT) to dissipate UV energy as heat, preventing polymer photodegradation. Their maximum absorption wavelength is around 260 nm, with a critical structure–property relationship: increased alkoxy substitution at the 4-position induces a red shift (longer absorption wavelength), extending the UV protection range [23,24,25].

The photostabilization mechanism of 2-hydroxybenzophenone is primarily governed by the intramolecular hydrogen bond between the H atom of the 2-hydroxy group and the O atom of the carbonyl group [26]. Upon absorbing ultra-violet light, 2-hydroxybenzophenone transitions to an excited state, where the H atom of the hydroxyl group is transferred to the O atom of the carbonyl group, forming an enol-quinone tautomer. 2-Hydroxybenzophenone absorbs ultra-violet light to reach its excited state, and the H on the hydroxyl group is transferred to the O atom of the carbonyl group, forming an enol-quinone structure. This tautomer is unstable and releases energy in the form of heat before rearranging back to the original structure, completing a cyclic process that mitigates the damaging effects of ultra-violet radiation [26].

Additionally, benzophenone has been demonstrated to function dually as a photoinitiator and ultra-violet absorber [27], capable of both initiating polymer cross-linking reactions and inhibiting light-induced polymer degradation [13,14,15,27]. The multifunctional benzophenone compounds used in the system reduce the chemical impurities of the polyethylene insulation. These voltage stabilizers have poor compatibility with polyethylene. Even if long alkyl chains are introduced into the core molecular structure, the migration and precipitation of doped voltage stabilizers from the polymer matrix cannot be avoided [28,29,30,31]. The migration will gradually lead to the formation of defects and loss of their initial effect.

Grafting voltage stabilizers onto polyethylene chains to form covalent bonds represents a promising solution to this issue. The reaction process of the ultra-violet radiation grafting of benzophenone compounds onto polyethylene remains poorly understood at the atomic or molecular level. Consequently, elucidating the chemical reaction pathway and evaluating the impacts of electron-donating and electron-withdrawing groups on the reaction potential barrier are critical for advancing the development of polyethylene insulation materials for high-voltage cables.

In this study, 4-methylheptane (Pe) was selected as the model molecule for polyethylene. The grafting reaction of benzophenone-type molecules was subjected to a comprehensive theoretical analysis utilizing density functional theory (DFT) [23] at both the atomic and molecular levels. Table 1 presents the molecular names, formulas, and corresponding abbreviations (abs.) of the molecules under investigation. The effects of electron-withdrawing groups (-NO_2_, -F, and -Cl) and electron-donating groups (-OH, -CH_3_, -NH_2_, and -OCH_3_) on the reaction potential barrier were evaluated to clarify substituent electronic effects on grafting reactivity.

## 2. Computation Methods

In this work, the geometry structure optimization and frequency calculation of the stationary points for the studied eleven reaction channels on the ground state S0 (or the triplet state T1) were carried out by density functional theory (DFT) [32] at the B3LYP/6-311+G(*d,p*) level [33,34,35,36]. B3LYP is a combination of Becke three-parameter mixed exchange functionals and Lee Yang Parr (LYP) related functionals, which is one of the most widely used methods in density functional theory (DFT). The core idea is to balance computational accuracy and cost by mixing different types of exchange functionals and related functionals, which is suitable for molecular geometry optimization, energy calculation, reaction mechanism research, and other scenarios [34].

(1) $$E_{XC}=a_{0}E_{X}^{Slater}+(1-a_{0})E_{X}^{HF}+a_{x}\Delta E_{X}^{Becke}+a_{c}E_{C}^{LYP}+\left(1-a_{c}\right)E_{c}^{VMN}$$

where $E_{X}^{Slater}$ is the local spin density exchange functional [37], $E_{X}^{HF}$ is the HartreeFock exact exchange functional, $\Delta E_{X}^{Becke}$ is the Becke gradient-corrected exchange functional [38], $E_{C}^{LYP}$ is the Lee, Yang, and Parr related functional [32], and $E_{c}^{VMN}$ is the related functional proposed by Vosko et al. [39]. $a_{0}$, $a_{x}$, and $a_{c}$ are semi-empirical coefficients representing the contributions of each term to the total exchange related functional, usually $a_{x}$ < $a_{0}$ < $a_{c}$. Becke gives the values of $a_{0}$, $a_{x}$, and $a_{c}$, which are 0.80, 0.72, and 0.81, respectively [36].

The potential energy surface information is obtained along the minimum energy path (MEP) by intrinsic reaction coordinate (IRC) theory with a gradient step-size of 0.05 (amu)^1/2^ Bohr. The first and second energy derivatives were obtained to calculate the curvature and the generalized vibrational frequencies along the reaction path. The values of *E*_g_, IP(a), and EA(a) were obtained based on the calculation results of the electronic structure. *E*_g_ refers to the energy gaps between the highest occupied molecular orbital (HOMO) and the lowest unoccupied molecular orbital (LUMO). IP(a) and EA(a) refer to the adiabatic ionization potentials and electron affinity energies, respectively. The time-dependent density functional theory (TDDFT) method [40,41] was employed to calculate the excitation energies of the design antioxidant molecules based on the optimized geometries at the same level. All electronic structure calculations were conducted using the GAUSSIAN 09 (Revision D.01) software package [42]. The relevant schematic formulas for ionization potential IPs and electron affinity EAs can be found in the reference literature [14]. A diagram showing the modification of geometric coordinates and the change in energy is also explained in the reference file [14].

## 3. Results and Discussion

### 3.1. Stationary Point Geometries

**Figure 1:**
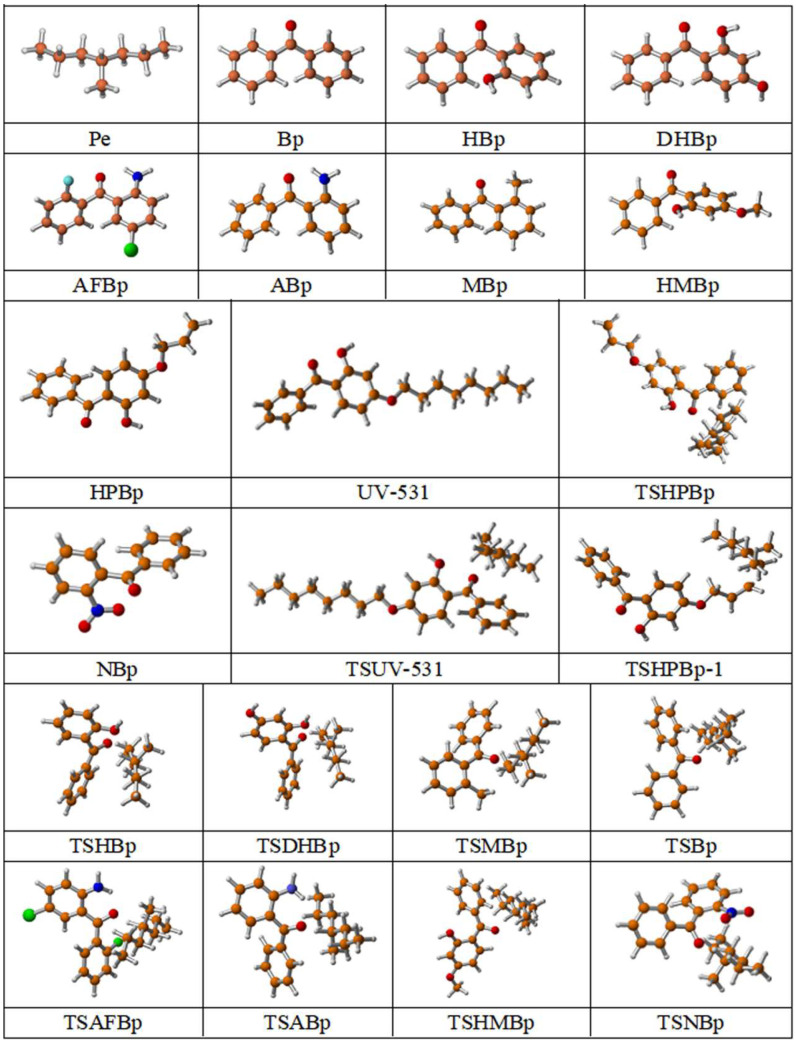
Optimized geometric structures of the studied molecules at the B3LYP/6-311+G(*d,p*) level, where orange represents C atoms, red represents O atoms, white represents H atoms, dark blue represents N atoms, sky blue represents F atoms, and green represents Cl atoms. (Supplementary Materials).

The optimized geometries of eleven reaction pathways at the B3LYP/6-311+G(*d*,*p*) level are illustrated in Figure 1 to facilitate comparison of structural variations among different reaction pathways. Table 2 presents the optimized bond lengths of the breaking and forming of C-H or O-H bonds in the corresponding reactants and the 11 transition states, as well as the resultant C-H bond lengths in the respective products. Furthermore, the calculated harmonic vibrational frequencies are included. This paper presents the chemical reaction equations for the grafting of biphenylmethane compounds onto polyethylene, which are potentially initiated by photoinitiators during the UV cross-linking of polyethylene.

Vibrational frequency analysis confirmed that all transition states exhibit exactly one imaginary frequency, corresponding to the coupled breaking of the original bond and stretching of the forming bond. All other stationary points were verified as true minima (i.e., no imaginary frequencies) via vibrational frequency analysis. Transition states are abbreviated as “TSs” for brevity.

Table 2 reveals that the transition state structures of the nine hydrogen abstraction reactions in the T1 state exhibit common structural characteristics, with the exception of the two reaction channels corresponding to TSAFBp and TSNBp. The elongation extents of the cleaving C-H bond in the model molecule Pe are smaller than those of the C-H bonds in the equilibrium structures of the corresponding diphenylmethanone compounds. This result indicates that these hydrogen abstraction reactions share similar characteristics and proceed via early transition states, aligning with the hallmark features of exothermic reactions, as postulated by Hammond [29].

### 3.2. Frontier MOs

The electron affinity energy (EA) and ionization potential (IP) of molecules are key parameters for evaluating their oxidation and reduction capabilities. Table 3 presents the calculated adiabatic electron affinity and adiabatic ionization potential IP(*a*) at the B3LYP/6-311+G(*d,p*) level of theory, along with the corresponding experimental data [30] and the calculated HOMO-LUMO energy gap (*E*_g_). Table 3 reveals that the adiabatic electron affinity EA(*a*) values of 2-hydroxybenzophenone derivatives are higher than that of the model molecule 4-methylheptane (−1.09 eV), while their adiabatic ionization potential IP(*a*) values are lower than that of 4-methylheptane (9.41 eV). When a substituent is incorporated onto the benzene ring of the benzophenone (Bp), the adiabatic electron affinity EA(*a*) of the derivative is modulated by the electronic nature of the substituent. The higher the electron density on the benzene ring, the weaker its electron-accepting ability and, consequently, the lower its adiabatic electron affinity EA(*a*) value. Therefore, when the benzene ring is connected at the 2-position with -OH, -CH_3_, and -NH_2_, respectively, these three groups are electron donor groups, which increases the density of the electron cloud on the benzene ring. Consequently, the adiabatic electron affinity EA(*a*) values of the derivatives (HBp 0.60 eV, MBp 0.67 eV, and ABp 0.67 eV) are lower than that of benzophenone (Bp 0.73 eV). For DHBp, HMBp, HPBp, and UV-531, there are two electron donor groups (on the 2-position and 4-position) attached to the benzene ring, which further reduces their values of EA(*a*), such as EA(*a*) (DHBp, 0.47 eV) < EA(*a*) (HBp, 0.60 eV), EA(*a*) (HMBp, 0.48 eV) < EA(*a*) (HBp, 0.60 eV). For the AFBp molecule, there are three substituent groups on the benzene ring: -NH_2_ on the 2-position is the electron donor group, while -F and -Cl are the electron-withdrawing groups on the 2′-position and 5-position, respectively. The electron cloud density of the molecule AFBp is less than that of Bp, according to the comprehensive effect, so the EA(*a*) value of AFBp increases, which is greater than that of Bp, EA(*a*) (AFBp, 0.89 eV) > EA(*a*) (Bp, 0.73 eV). For NBp, when the strong electron-withdrawing group -NO_2_ is substituted at the 2-position of the benzene ring, this substitution significantly reduces the electron density of the benzene ring, making it lower than the electron cloud density of other molecules studied, and consequently yields the highest adiabatic electron affinity EA(*a*) value (1.62 eV) in Table 3. According to the findings of Chen and co-workers, the stronger the electron-withdrawing groups, the greater the electron affinity and energy gap, and the better the electrical resistance, while the addition of the electron donor group will lead to a smaller energy gap [43]. The *E*_g_ value of NBp is relatively low, and the EA(*a*) value is the highest, so it is concluded that the electrical resistance performance as the voltage stabilizer is relatively high, which is consistent with the research result obtained by Jarvid et al. [22].

### 3.3. Energetics

Table 2 presents the reaction Gibbs free energy (ΔG) at 298 K and reaction Gibbs potential barrier heights (ΔG^≠^) for the eleven reaction pathways. Upon excitation, benzophenone undergoes a transition from the ground state S0 to the singlet excited state S1 (n, π*), followed by intersystem crossing (ISC) to the excited triplet state T1 (n, π*). Stéphane and co-workers elucidated the mechanism underlying the S1 → T1 transition in benzophenone by analyzing sub-picosecond time-resolved absorption spectra across multiple solvents and excitation wavelengths, using 4-methoxybenzophenone (4-MeOBP) as a model compound [44]. Granucci and colleagues simulated the photodynamic processes of benzophenone (Bp) within the first 20 ps, considering kinetic effects and spin–orbit coupling to clarify the S1 → T1 decay mechanism [45]. They proposed that intersystem crossing (ISC) is the primary channel for S1 → T1 transitions. Zhang and co-workers calculated the potential barrier of 0.17 eV for the UV-induced cross-linking of polyethylene initiated by benzophenone using density functional theory (DFT) calculations [15]. Benzophenone-derived radicals can graft onto polyethylene chains, yielding a novel polyethylene-based material with ultra-violet (UV) resistant properties.

Additionally, benzophenone compounds can function as both UV absorbers and voltage stabilizers, which not only reduce the electronic conductivity of the material but also lower additive dosage, thereby minimizing impurity incorporation.

In the context of benzophenone grafting onto polyethylene, the greater the electron deficiency of the C on carbonyl, the higher its electropositivity, and the stronger its attraction to unpaired electrons. Conversely, the stronger the electron-donating ability of substituents attached to the benzene ring, the lower the electropositivity of the carbonyl carbon. Furthermore, if the carbonyl O participates in intramolecular hydrogen bonding, the electron density of the C=O bond will further shift toward O, thereby enhancing the electropositivity of the carbonyl C.

For reaction channels ① and ⑤, the 2-position of the benzene ring is connected to a methyl group, and the intramolecular hydrogen bond cannot be formed because there is no active hydrogen. Methyl is the electron donor group, which will slightly increase the density of the electron cloud on the benzene ring, thus slightly reducing the positive electricity of C on the carbonyl. Therefore, MBp reactivity is slightly lower than Bp, which is consistent with the calculated results of the reaction Gibbs potential barrier heights ΔG^≠^_MBp_ (0.80 eV) > ΔG^≠^_Bp_ (0.71 eV).

For reaction channels ①, ②, and ⑥, the hydroxyl and amino groups are connected at the 2-position of the benzene ring. -OH and -NH_2_ are strong electron donors, which greatly reduces the positive electricity of C on the carbonyl. The electron-donating ability of -NH_2_ is stronger than that of -OH. The active H atoms in -OH and -NH_2_ can form hydrogen bonds with O on the carbonyl, which increases the positive electricity of C on the carbonyl. Because the electronegativity of O is higher than that of N, the activity of the H atom on -OH is higher, and it is easier to form a hydrogen bond with the carbonyl O atom, so the ability of -OH to form hydrogen bonds is stronger than that of -NH_2_. Considering these two factors comprehensively, it can be seen that the influence of the hydrogen bond force of -OH and -NH_2_ is weaker than the electron-donating effect, and the positive electricity of C on the carbonyl decreases. Therefore, the reaction Gibbs potential barrier heights are ΔG^≠^_Bp_ (0.71 eV) < ΔG^≠^_HBp_ (0.72 eV) < ΔG^≠^_ABp_ (1.05 eV).

For reaction channels ①, ④, ⑦, ⑧, ⑨, and ⑩, in DHBp, HMBp, HPBp, and UV-531, the hydroxyl groups are connected at the 2-position of the benzene ring, which is consistent with HBp. In DHBp, HMBp, HPBp, and UV-531. -OR is also connected at the 4-position of the benzene ring. The -OR group only reflects a strong electron donor effect, which reduces the positive charge of the carbonyl C. Therefore, the reactivity of DHBp, HMBp, HPBp, and UV-531 is lower than that of Bp; that is, ΔG^≠^_DHBp_ (0.75 eV), ΔG^≠^_HMBp_ (0.79 eV), ΔG^≠^_HPBp_ (0.78 eV), and ΔG^≠^_UV-531_ (0.80 eV) > ΔG^≠^_Bp_ (0.71 eV). The UV absorber HPBp, which contains an unsaturated graftable vinyl group with a long alkyl chain, can be used as a high-efficiency voltage stabilizer. It was reported by Li and co-workers that the grafting reaction of voltage stabilizer HPBP onto polyethylene took place on the unsaturated vinyl group of HPBP, avoiding the migration and precipitation of the voltage stabilizer and improving the electrical resistance of polyethylene [27]. The calculated reaction Gibbs potential barrier height of the unsaturated graftable vinyl group (reaction channel ⑨) is higher than that of the reaction channel ⑧ in this work; ⑧ is the dominant reaction channel. It is verified that the carbonyl group is the reactive site of the hydrogen extraction reaction.

For reaction channels ① and ③, in AFBp, compared with ABp, there is one more F atom at the 2′-position. The electronegativity of the F atom is the strongest among all elements, and the conjugation effect is relatively smaller than that of the O atom and the N atom. Therefore, it shows a very strong electron-withdrawing effect. The distance between 2′-F and the carbonyl O atom is relatively close. Both induction and field effects will transfer the electron cloud from O to the C atom [26], thus reducing the positive charge of the carbonyl and the reaction activity. Thus, ΔG^≠^_AFBp_ (1.12 eV) > ΔG^≠^_Bp_ (0.71 eV).

For reaction channels ① and ⑪, in NBp, a single benzene ring electron undergoes sp^2^ hybridization to form a large π bond, resulting in consistent bond length and energy. When -NO_2_ is introduced, the oxygen atom on the -NO_2_ group is more attractive to electrons. The -NO_2_ group is an electron-withdrawing group, which greatly reduces the π electron density on the benzene ring and, at the same time, increases the electron cloud density of C on the carbonyl group connected to the benzene ring, thus reducing the positive electricity and reaction activity of the carbonyl group. Thus, ΔG^≠^_NBp_ (0.80 eV) > ΔG^≠^_Bp_ (0.71 eV).

## 4. Conclusions

The reaction mechanism of benzophenone-grafted polyethylene was systematically investigated via theoretical approaches. Benzophenone compound photoinitiators, functioning as stabilizers, can be covalently grafted onto polyethylene chains. This grafting strategy effectively mitigates the migration and precipitation of benzophenone compounds from the polymer matrix. Among the derivatives studied, NBp exhibits the highest electron affinity (EA) due to the introduction of the strong electron-withdrawing group (-NO_2_). The information regarding the potential energy surfaces of the eleven reaction channels would facilitate the development of a theoretical basis for the rational design of stabilizer molecules and the optimization of UV-irradiation cross-linking processes.

## Figures and Tables

**Table 1:** The molecular name, molecular formula, and corresponding abbreviation (ab.) of the studied molecules.

Molecular Formula	Molecular Name	ab.	Molecular Formula	Molecular Name	ab.
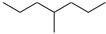	4-Methylheptane	Pe	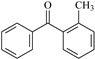	2-Methylbenzophenone	MBp
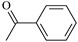	Acetophenone	Ap	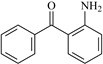	2-Aminobenzophenone	ABp
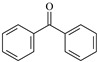	Benzophenone	Bp	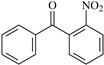	2-Nitrobenzophenone	NBp
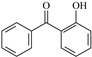	2-Hydroxybenzophenone	HBp	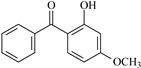	2-Hydroxy-4-methoxybenzophenone	HMBp
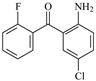	2-Amino-2’-fluoro-5-chlorobenzophenone	AFBp	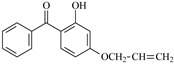	2-Hydroxy-4-(2-propenyloxy)lbenzophenone	HPBp
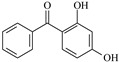	2,4-Dihydroxybenzophenone	DHBp	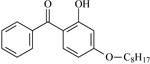	2-Hydroxy-4-octyloxybenzophenone	UV-531

**Table 2:** Optimized bond lengths of breaking/forming bonds for the transition state (b/f), reactants, and products (in angstrom), together with the calculated breaking/forming bond frequencies (in cm^−1^), the reaction Gibbs free energy (ΔG), and the potential barrier heights (ΔG^≠^) (in eV).

Reaction Equation			B3LYP/6-311+G(*d*,*p*)					
			ΔG^≠^	ΔG	Reactant	b/f	Product	Freq.
①	TSBp	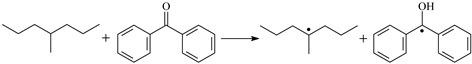	0.71	−0.46	1.100	1.228/1.395	0.964	835 *i*
②	TSHBp	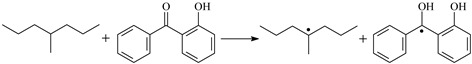	0.72	−0.60	1.100	1.224/1.407	0.965	1019 *i*
③	TSAFBp	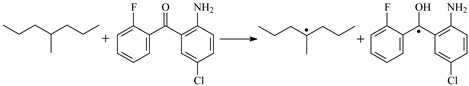	1.12	0.06	1.100	1.247/1.371	0.983	1151 *i*
④	TSDHBp	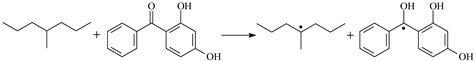	0.75	−0.36	1.100	1.239/1.375	0.965	1156 *i*
⑤	TSMBp	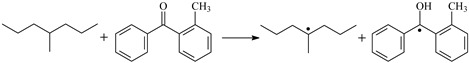	0.80	−0.38	1.100	1.251/1.362	0.965	1149 *i*
⑥	TSABp	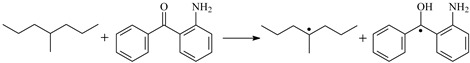	1.05	−0.08	1.100	1.262/1.349	0.965	1331 *i*
⑦	TSHMBp	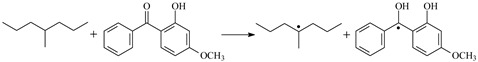	0.79	−0.36	1.100	1.251/1.354	0.965	1181 *i*
⑧	TSHPBp	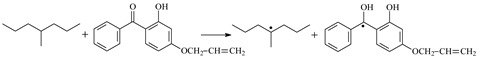	0.78	−0.34	1.100	1.247/1.358	0.963	1149 *i*
⑨	TSHPBp-1	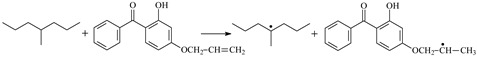	0.99	−0.49	1.100	1.329/1.420	1.092	1654 *i*
⑩	TSUV-531	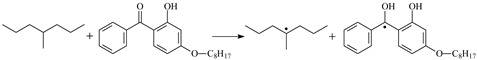	0.80	−0.34	1.100	1.252/1.349	0.965	1216 *i*
⑪	TSNBp	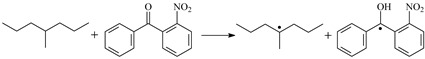	0.80	0.33	1.100	1.222/1.442	1.018	537 *i*

**Table 3:** The *E*_g_, IP, and EA of studied molecules as well as the corresponding experimental data in brackets (in eV).

Molecular Formula	ab.	*E*_g_	EA (*a*)	IP (*a*)	EA (*v*)	IP (*v*)
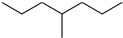	Pe	8.38	−1.09	9.41	−1.10	10.03
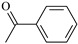	Ap	5.20	0.33 (0.33)	8.95 (9.1 ± 0.1)	0.09	9.19
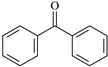	Bp	4.90	0.73 (0.69 ± 0.05)	8.52 (9.05)	0.50	8.67
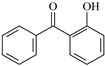	HBp	4.73	0.60	8.14	0.35	8.38
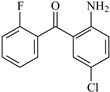	AFBp	3.91	0.89	7.60	0.60	7.81
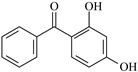	DHBp	4.63	0.47	7.83	0.24	8.17
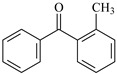	MBp	4.89	0.67	8.30	0.46	8.55
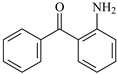	ABp	3.98	0.67	7.51 (8.3 ± 0.1)	0.37	7.70
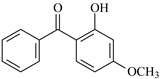	HMBp	4.62	0.48	7.73	0.23	8.05
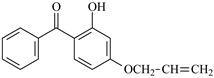	HPBp	4.59	0.46	7.62	0.24	8.00
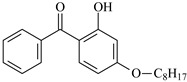	UV-531	4.49	0.41	7.50	0.19	7.84
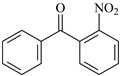	NBp	4.46	1.62	8.85	1.12	9.05

## Data Availability

The original contributions presented in this study are included in the article/Supplementary Material. Further inquiries can be directed to the corresponding author.

## Supplementary Materials

The following supporting information can be downloaded at: https://www.mdpi.com/article/10.3390/polym17192595/s1, Supplementary S1: Optimized geometric structures of the studied molecules at the B3LYP/6-311+G(*d*,*p*) level.
