# Supplementary material for: Theoretical Study on the Grafting Reaction of Benzophenone Compounds to Polyethylene in the UV Radiation Cross-Linking Process
Source: Polymers (Basel). 2025 Sep 25;17(19):2595. doi: 10.3390/polym17192595 (PMC12526467; doi:10.3390/polym17192595)
Supplement: Supplementary file 1 [file polymers-17-02595-s001.zip › polymers-3858906-supplementary.pdf]

Theoretical Study on the Grafting Reaction of Benzophenone Compounds to Polyethylene in the UV Radiation Cross-Linking Process

Yang Du, Chi Deng, Hui Zhang *, Xia Du, Yan Shang and Xuan Wang

Key Laboratory of Engineering Dielectrics and Its Application of Ministry of Education, School of Material Science and Chemical Engineering, Harbin University of Science and Technology, Harbin 150080, China; duyang950711@163.com (Y.D.); dengchi@potevio.com (C.D.); duxia62@126.com (X.D.); shangyan1972@126.com (Y.S.); wangxuan@hrbust.edu.cn (X.W.)
* Correspondence: huizhang@hrbust.edu.cn

Optimized geometric structures of the studied molecules at the B3LYP/6-311+G(*d,p*) level.

PE

C	0.26356000	−0.70889000	3.78958200
H	−0.70611700	−1.20475700	3.90024400
H	1.02170900	−1.49016800	3.67484900
H	0.47239400	−0.17768400	4.72249600
C	0.26356000	0.24421600	2.58999800
H	−0.47536800	1.03361400	2.76189600
H	1.23729600	0.74550900	2.52320700
C	−0.02580600	−0.47016600	1.26348000
H	0.68391100	−1.30037000	1.15669500
H	−1.02292600	−0.92984000	1.31031400
C	0.06115200	0.40922400	0.00000000
H	1.05108500	0.88891200	0.00000000
C	−0.02580600	−0.47016600	−1.26348000
H	−1.02292600	−0.92984000	−1.31031400
H	0.68391100	−1.30037000	−1.15669500
C	0.26356000	0.24421600	−2.58999800
H	1.23729600	0.74550900	−2.52320700
H	−0.47536800	1.03361400	−2.76189600
C	0.26356000	−0.70889000	−3.78958200
H	−0.70611700	−1.20475700	−3.90024400
H	0.47239400	−0.17768400	−4.72249600
H	1.02170900	−1.49016800	−3.67484900
C	−1.00215100	1.51849000	0.00000000
H	−0.91638400	2.16177700	−0.87875400
H	−2.00987800	1.08671800	0.00000000
H	−0.91638400	2.16177700	0.87875400

Bp

C	−2.68840400	1.51075900	−0.72551700

C	-1.44193600	0.89090800	-0.66343400
C	-1.29976800	-0.34841500	-0.02604400
C	-2.42628600	-0.95695200	0.54455400
C	-3.66392300	-0.32599900	0.50257600
C	-3.79778000	0.90898300	-0.13471300
H	-2.79271300	2.46149300	-1.23595100
H	-0.58564100	1.35925800	-1.13318400
H	-2.31122000	-1.92643100	1.01391300
H	-4.52687500	-0.79724700	0.95934400
H	-4.76526100	1.39694800	-0.17494500
C	-0.00000600	-1.09820800	0.00004600
C	1.29977600	-0.34845500	0.02612400
C	2.42624600	-0.95695600	-0.54460900
C	1.44199400	0.89083600	0.66356800
C	3.66387800	-0.32599000	-0.50272400
H	2.31113900	-1.92640700	-1.01401700
C	2.68845800	1.51069900	0.72555900
H	0.58574400	1.35914100	1.13344200
C	3.79778200	0.90896500	0.13460900
H	4.52679300	-0.79720300	-0.95959800
H	2.79281100	2.46140100	1.23604100
H	4.76526100	1.39694000	0.17477000
O	-0.00002800	-2.31861700	0.00002700

HBp

C	2.64675100	1.36003300	-1.05523400
C	1.42811400	0.73664400	-0.80126300
C	1.37652300	-0.41622700	-0.00970300
C	2.56282900	-0.93497300	0.52585000
C	3.77620500	-0.30029000	0.28895900
C	3.82059100	0.84718400	-0.50513000
H	2.68115300	2.24496100	-1.68066700
H	0.51776500	1.13788800	-1.23038700
H	2.51120000	-1.83584100	1.12496200
H	4.68870500	-0.69965400	0.71711900
H	4.76844300	1.33810900	-0.69646700
C	0.10168800	-1.16215800	0.22661100
C	-1.22535400	-0.47725100	0.02929000
C	-1.56585500	0.74346000	0.63361900
C	-2.19593100	-1.13852600	-0.73255900
C	-2.83663800	1.29190300	0.44258000
C	-3.45667800	-0.59003100	-0.93789900
H	-1.93667100	-2.09800300	-1.16424600
C	-3.77321600	0.63222200	-0.34636600

H	-3.09206400	2.23162400	0.92403200
H	-4.18657000	-1.11161200	-1.54492500
H	-4.75396900	1.07162400	-0.48904000
O	0.11969600	-2.34472700	0.52397100
O	-0.63801400	1.34661300	1.43559900
H	-1.00560800	2.15387200	1.81053300

DHBp

C	3.04199500	-1.54073000	-1.02681200
C	1.84994400	-0.86923800	-0.76160200
C	1.83540500	0.23099900	0.10469200
C	3.03460000	0.64955600	0.69786100
C	4.21947300	-0.03148800	0.44644500
C	4.22569300	-1.12802900	-0.41830200
H	3.04654800	-2.38305000	-1.70943100
H	0.93368700	-1.18782400	-1.24352200
H	3.01427400	1.51411400	1.35024600
H	5.14041900	0.29230000	0.91823700
H	5.15172300	-1.65514900	-0.61969300
C	0.59129900	1.03086600	0.37528400
C	-0.74198800	0.36386600	0.20914400
C	-1.82462700	1.01750300	-0.41590300
C	-0.97816200	-0.89429100	0.77224200
C	-3.08157300	0.41773100	-0.46511900
C	-2.23188800	-1.49412400	0.75062000
H	-0.15933600	-1.40808300	1.26209200
C	-3.28673200	-0.82778500	0.12428400
H	-3.91376100	0.90919700	-0.95807900
H	-2.38827500	-2.46244400	1.21441800
O	0.67349300	2.18688000	0.74795500
O	-1.59726700	2.22083000	-1.00322900
H	-2.41031100	2.54668300	-1.40346500
O	-4.54646900	-1.34680800	0.04802700
H	-4.57366500	-2.20198100	0.49017900

AFBp

C	2.99081200	-1.39926500	1.46219600
C	1.88195700	-0.56261000	1.36454400
C	1.69384400	0.25589100	0.24508600
C	2.64869200	0.19350800	-0.76683200
C	3.75247600	-0.64210100	-0.70152300
C	3.92421200	-1.43967800	0.42747100
H	3.12411000	-2.01763700	2.34168700
H	1.15507000	-0.53022000	2.16839200

H	4.45555900	-0.65662700	-1.52506600
H	4.78591600	-2.09338000	0.49459300
C	0.56346200	1.25742100	0.18415700
C	-0.82512700	0.77265900	0.12162500
C	-1.92356000	1.68248600	0.21855400
C	-1.08959300	-0.60036100	-0.06879700
C	-3.23376800	1.15583600	0.14693500
C	-2.38078700	-1.07411700	-0.14295800
H	-0.26214800	-1.28907100	-0.16749300
C	-3.46245500	-0.19290100	-0.02977800
H	-4.07598100	1.83488100	0.22818200
H	-4.47576800	-0.57077200	-0.08645400
O	0.85205300	2.45144800	0.21370200
F	2.48505400	0.95483000	-1.87225800
Cl	-2.68298900	-2.79225300	-0.38536400
N	-1.74076000	3.01758100	0.39620300
H	-2.53288200	3.63653500	0.37119300
H	-0.80063800	3.38587300	0.32935600

ABp

C	-3.04860900	-1.26436400	0.93104000
C	-1.74604600	-0.77851700	0.83524500
C	-1.45436700	0.30108600	-0.00744600
C	-2.48969300	0.89026900	-0.74438100
C	-3.78383000	0.38685600	-0.66844900
C	-4.06676300	-0.69084800	0.17174500
H	-3.26748700	-2.08900500	1.60004100
H	-0.96095000	-1.22288400	1.43516200
H	-2.26499000	1.74545600	-1.37026600
H	-4.57456300	0.83933800	-1.25627200
H	-5.07790600	-1.07647100	0.23949700
C	-0.08997700	0.93150900	-0.07046100
C	1.12331500	0.08833700	-0.10345900
C	2.40481900	0.64535100	0.19896400
C	1.05442300	-1.26500700	-0.49379200
C	3.53308400	-0.20310700	0.15663400
C	2.17571600	-2.07248100	-0.55866300
H	0.09245400	-1.67396000	-0.77344100
C	3.42028900	-1.52922700	-0.21357800
H	4.50451000	0.21113200	0.40733300
H	2.09227400	-3.10490500	-0.87449300
H	4.31047300	-2.14815400	-0.24783900
O	-0.02775100	2.16304800	-0.08842400
N	2.55695600	1.95263500	0.56113700

H	1.78801100	2.58194700	0.37173600
H	3.48731700	2.32554700	0.64757400

MBp

C	2.88031400	-1.32284700	1.03033500
C	1.61073700	-0.81409700	0.76367300
C	1.45338700	0.27553100	-0.10259700
C	2.58838100	0.84734700	-0.69479200
C	3.85163900	0.32591000	-0.44391400
C	4.00020700	-0.76000200	0.42143200
H	2.99431000	-2.15737600	1.71289400
H	0.74459200	-1.25215800	1.24422700
H	2.45705200	1.70225700	-1.34692200
H	4.72241700	0.76603300	-0.91664800
H	4.98679700	-1.16245500	0.62272400
C	0.11848900	0.91155000	-0.36597300
C	-1.13170000	0.09092000	-0.22402500
C	-2.29681000	0.63583100	0.36086000
C	-1.15900600	-1.20376100	-0.76068300
C	-3.44383000	-0.16310300	0.39915900
C	-2.32167500	-1.96717400	-0.74028200
H	-0.26319600	-1.60665800	-1.21852800
C	-3.46757500	-1.44400800	-0.14844800
H	-4.34050400	0.23305100	0.86440600
H	-2.32941700	-2.95936000	-1.17643500
H	-4.37948300	-2.02966400	-0.11060600
O	0.05773600	2.08316700	-0.70199000
C	-2.34592000	2.02641300	0.94754700
H	-1.46856500	2.24485500	1.56005800
H	-2.36874000	2.78197300	0.15926500
H	-3.23698200	2.14310000	1.56773900

HMBp

C	3.26652000	1.49213200	-1.10813400
C	2.10467600	0.78131200	-0.82005200
C	2.16341500	-0.38899800	-0.05557300
C	3.40339400	-0.83662200	0.41788300
C	4.56034400	-0.11419800	0.14953700
C	4.49436200	1.05045100	-0.61721800
H	3.21457600	2.38974900	-1.71409500
H	1.15210100	1.12746400	-1.20286800
H	3.43918900	-1.75327100	0.99380800
H	5.51466400	-0.45921700	0.53142400
H	5.39800300	1.60954900	-0.83390500

C	0.95320200	-1.23270700	0.20333400
C	-0.42008800	-0.63188600	0.15044500
C	-0.78947500	0.57992000	0.76743300
C	-1.42514400	-1.36360000	-0.48828200
C	-2.09999600	1.03936800	0.70018900
C	-2.73762900	-0.91469100	-0.57985200
H	-1.15248900	-2.31492300	-0.92929700
C	-3.07500100	0.30325900	0.02073000
H	-2.39263200	1.96406800	1.18655900
H	-3.47275200	-1.50905800	-1.10350600
O	1.07435200	-2.43286100	0.39594700
O	0.16154900	1.26362600	1.46867800
H	-0.22820400	2.04950400	1.86623500
O	-4.31843300	0.85263800	0.00983600
C	-5.37262100	0.14831100	-0.63921800
H	-5.54340600	-0.82782200	-0.17438200
H	-6.25943000	0.76749800	-0.51706900
H	-5.16512500	0.01691800	-1.70592600

HPBp

C	3.62313500	-2.07365100	-1.04242000
C	2.65779400	-1.10396600	-0.77789100
C	2.93155500	-0.05720100	0.11120900
C	4.18873500	0.00369600	0.72845100
C	5.14441300	-0.97333900	0.47743500
C	4.86360700	-2.01418200	-0.41036000
H	3.40758500	-2.87287300	-1.74265500
H	1.69776000	-1.14894700	-1.27733700
H	4.39568300	0.82904300	1.39888900
H	6.11028900	-0.92443000	0.96762300
H	5.61190200	-2.77284300	-0.61157300
C	1.95087300	1.04994800	0.38347100
C	0.48944800	0.78119900	0.18140800
C	-0.35895600	1.72038100	-0.44493100
C	-0.09829600	-0.37006700	0.70867100
C	-1.72739200	1.48972200	-0.52492600
C	-1.47010600	-0.60693100	0.65715700
H	0.53586800	-1.10009100	1.19831000
C	-2.29030400	0.33670100	0.03162900
H	-2.38267300	2.19951400	-1.01903400
H	-1.87389200	-1.50713600	1.09767300
O	2.34195400	2.13066100	0.78541400
O	0.20467900	2.82515500	-1.00145200
H	-0.47898000	3.36778700	-1.40825500

O	-3.64001600	0.22701200	-0.09902200
C	-4.30027000	-0.91473600	0.46585200
H	-4.07226800	-0.96484500	1.53870300
H	-3.94028000	-1.83382400	-0.00972400
C	-5.77227900	-0.74606100	0.25630000
H	-6.19574800	0.18215100	0.63094200
C	-6.54929900	-1.65795300	-0.31997100
H	-7.61837400	-1.51015400	-0.41932300
H	-6.14575200	-2.58734800	-0.71026300

UV-531

C	-6.42190600	-1.98042500	-1.14571800
C	-5.38113600	-1.10822900	-0.83193600
C	-5.61514500	0.00987700	-0.02200700
C	-6.90869600	0.24095100	0.46576400
C	-7.94202200	-0.63905300	0.16707400
C	-7.70050700	-1.75196100	-0.64108600
H	-6.23468500	-2.83638600	-1.78436200
H	-4.39058400	-1.28601500	-1.23250800
H	-7.08152000	1.12013400	1.07460800
H	-8.93696000	-0.45837500	0.55834300
H	-8.50794100	-2.43558400	-0.87924400
C	-4.54538600	1.02013900	0.29291000
C	-3.11388900	0.57997900	0.26955500
C	-2.09952200	1.38737800	-0.27233600
C	-2.72566700	-0.61898300	0.88944300
C	-0.75656900	1.00095800	-0.19507600
C	-1.40157500	-1.00520200	0.99509300
H	-3.49201300	-1.25117600	1.32225500
C	-0.40398200	-0.18866300	0.44603200
H	-0.00285100	1.63958800	-0.63950000
H	-1.11509600	-1.92181600	1.49427000
O	-4.85221100	2.16235600	0.58452700
O	-2.45478700	2.53272700	-0.91419600
H	-1.66863500	2.97438800	-1.25141600
O	0.87167600	-0.63498500	0.57760400
C	1.95260400	0.15101300	0.06514900
H	1.83040200	0.28426600	-1.01733000
H	1.94255600	1.14117200	0.53789000
C	3.24967500	-0.57934800	0.37398400
H	3.21787000	-1.56697800	-0.09792000
H	3.30733200	-0.74687600	1.45453900
C	4.48430700	0.19392400	-0.10390600
H	4.40684300	0.38084500	-1.18273900

H	4.50722600	1.18030900	0.37669700
C	5.80051900	-0.53724200	0.18560700
H	5.87366100	-0.73511700	1.26247700
H	5.78261600	-1.51931200	-0.30354000
C	7.04289000	0.23486000	-0.27247000
H	6.96723400	0.43762600	-1.34868700
H	7.06155200	1.21528600	0.22079900
C	8.35871800	-0.49815700	0.01165600
H	8.43238300	-0.70666900	1.08698200
H	8.34337400	-1.47640700	-0.48626700
C	9.60368600	0.27565800	-0.43723800
H	9.52990600	0.48545000	-1.51133800
H	9.62089100	1.25192800	0.06233600
C	10.91284600	-0.46605600	-0.15188300
H	10.93996400	-1.43225700	-0.66553500
H	11.78003200	0.11092000	-0.48490500
H	11.03354700	-0.65823000	0.91896000

NBp

C	-2.37719800	2.39673400	0.16414800
C	-1.17131700	1.84895600	0.59473300
C	-0.90788100	0.48395900	0.45154300
C	-1.89985700	-0.30331200	-0.13759500
C	-3.11561800	0.22425800	-0.56369200
C	-3.35160700	1.58528500	-0.41557400
H	-2.55687400	3.45859800	0.28645600
H	-0.42515400	2.48290200	1.05996900
H	-3.84935500	-0.43633000	-1.00487600
H	-4.29070100	2.00877000	-0.75027100
C	0.37184400	-0.05886500	1.07549400
C	1.65323000	0.08467000	0.32997800
C	2.84446900	-0.27268200	0.97681200
C	1.69925100	0.55667000	-0.98710500
C	4.06053300	-0.15400300	0.31655000
H	2.79179600	-0.64282100	1.99334700
C	2.91909000	0.67272100	-1.64808300
H	0.78357700	0.82734100	-1.49925800
C	4.09968100	0.31920000	-0.99696000
H	4.97922800	-0.43177700	0.82041800
H	2.94844500	1.03429200	-2.66946100
H	5.04956700	0.40905800	-1.51210300
O	0.31395300	-0.49226600	2.20786100
N	-1.66846100	-1.74973700	-0.34540300
O	-0.51211400	-2.14800800	-0.28383700

O	-2.63921700	-2.45515400	-0.58251300

TSHPBp

C	-3.07119800	0.86868000	-3.66111400
H	-4.07888800	0.74218600	-4.07100200
H	-3.03468200	1.84228600	-3.16341200
H	-2.37327200	0.89942000	-4.50251600
C	-2.71853200	-0.26145600	-2.68867200
H	-2.71302200	-1.21275500	-3.23072500
H	-1.70170700	-0.11649400	-2.31098100
C	-3.69568700	-0.33666500	-1.50887600
H	-3.78979200	0.65696500	-1.05665500
H	-4.69948900	-0.58302900	-1.89395900
C	-3.37423000	-1.34439200	-0.40615900
H	-2.29527200	-0.97928200	0.10215700
C	-4.39750700	-1.30381100	0.73004000
H	-5.34329600	-1.71024700	0.33606200
H	-4.60897400	-0.25667200	0.97536300
C	-4.03069600	-2.05641600	2.01669200
H	-3.05852700	-1.70352200	2.37634700
H	-3.90840400	-3.12302400	1.80416800
C	-5.08472600	-1.88263700	3.11472200
H	-6.06163700	-2.25774200	2.79256000
H	-4.80457400	-2.42433000	4.02225700
H	-5.20851200	-0.82854400	3.38300600
C	-3.02422800	-2.74326500	-0.89319000
H	-2.75528200	-3.40330900	-0.06740500
H	-3.88823500	-3.18397600	-1.41083100
H	-2.18450300	-2.72680600	-1.58834200
C	-0.94052800	3.90989200	-0.13809300
C	-0.35348800	2.65755400	-0.21246400
C	-0.95536400	1.52638500	0.40374200
C	-2.17995300	1.73794100	1.09184300
C	-2.75507200	2.99841500	1.16140800
C	-2.14764500	4.09762400	0.54724500
H	-0.46181500	4.75169500	-0.62742100
H	0.56828700	2.52923700	-0.76731200
H	-2.64358000	0.90465200	1.60352500
H	-3.68124400	3.13164700	1.71109100
H	-2.60226400	5.07978000	0.60181100
C	-0.35939000	0.22544300	0.32742300
C	1.07826300	-0.01478200	0.19618200
C	1.60492700	-1.05538900	-0.61749200

C	2.00994500	0.74828300	0.92270500
C	2.97184100	-1.28686900	-0.68182500
C	3.38139600	0.53608400	0.85623400
H	1.63240400	1.53207200	1.56807700
C	3.86751100	-0.50130200	0.05157500
H	3.37216600	-2.07231500	-1.31490600
H	4.04582400	1.15999700	1.43736700
O	-1.03650500	-0.90332200	0.60692500
O	0.73833800	-1.78393000	-1.37755800
H	1.23717300	-2.41619700	-1.90590800
O	5.18234700	-0.82484100	-0.09302500
C	6.16099900	-0.09642200	0.66045700
H	5.92184700	-0.17691300	1.72914900
H	6.14543800	0.96267100	0.37986000
C	7.50086500	-0.70229600	0.38268400
H	7.57477700	-1.77335900	0.55218800
C	8.56151100	-0.00814800	-0.01817400
H	9.52381500	-0.48242500	-0.17229900
H	8.50772400	1.06038500	-0.20339200

TSHPBp-1

C	4.09842900	-0.74472300	3.91647100
H	4.27666800	0.13267200	4.54614000
H	3.01532100	-0.85476900	3.80303800
H	4.46552400	-1.62122200	4.45713300
C	4.78878300	-0.59948100	2.55633300
H	5.87018900	-0.53135100	2.71125300
H	4.61972300	-1.50556200	1.96426100
C	4.29083500	0.62471100	1.77493600
H	3.19825400	0.56363000	1.68936500
H	4.48370600	1.52687300	2.37899700
C	4.88085400	0.85241100	0.38296000
C	4.14876700	1.97317800	-0.35564400
H	4.31775100	2.91329900	0.19548700
H	3.06837500	1.79084200	-0.28957300
C	4.51719600	2.20070900	-1.82848400
H	4.37501600	1.27081100	-2.38984900
H	5.57891900	2.45248800	-1.91465400
C	3.68677000	3.31558000	-2.47267100
H	3.83108800	4.26750200	-1.95191900
H	3.96490000	3.46472200	-3.51949900
H	2.61759400	3.08263600	-2.44358700
C	6.39703500	0.97565800	0.36073000
H	6.78603200	1.04467100	-0.65744200

H	6.71970800	1.87924700	0.89750000
H	6.88223200	0.12179100	0.83838500
C	-4.98877500	2.82366500	-0.60626100
C	-4.43229700	1.54729800	-0.54540500
C	-4.89810200	0.61638000	0.39132900
C	-5.93158000	0.98429800	1.26393300
C	-6.47369000	2.26291000	1.21428700
C	-6.00384400	3.18553600	0.27711200
H	-4.63189700	3.53335600	-1.34421400
H	-3.64943600	1.26760900	-1.23985800
H	-6.29720800	0.24901300	1.97047300
H	-7.26537400	2.54208300	1.90052600
H	-6.43167800	4.18105800	0.23369400
C	-4.38169600	-0.79491800	0.45578200
C	-2.99698000	-1.07777100	-0.04307800
C	-2.70374800	-2.22519900	-0.81265000
C	-1.92710200	-0.26582000	0.33889800
C	-1.39239800	-2.51996000	-1.16648300
C	-0.60498800	-0.55803000	0.01228800
H	-2.13103400	0.61560900	0.93578600
C	-0.33830900	-1.69963300	-0.75040400
H	-1.16281700	-3.39165400	-1.77054000
H	0.18670000	0.09368500	0.35266500
O	-5.07251200	-1.67775300	0.93146500
O	-3.73866200	-3.00139100	-1.23051000
H	-3.40162700	-3.73536800	-1.75493900
O	0.89889900	-2.09785400	-1.14485700
C	2.03519300	-1.31943300	-0.75879200
H	2.07368000	-1.24372300	0.34017100
H	1.92469100	-0.29203900	-1.14340400
C	3.25610000	-1.97471200	-1.29968000
H	3.10236900	-2.88420800	-1.87480000
C	4.61559000	-1.45298400	-1.07338600
H	4.64521500	-0.25799400	-0.30740800
H	5.26680100	-2.10135400	-0.47760000
H	5.14654500	-1.13636700	-1.97710300

TSUV-531

C	7.02413100	-2.43589000	2.97806400
H	7.81523500	-3.10093300	2.61660000
H	7.44533300	-1.42767900	3.04362000
H	6.76273000	-2.75171100	3.99170500
C	5.80281100	-2.46926800	2.05376200
H	5.38536500	-3.48108900	2.05057300

H	5.01856700	-1.81672300	2.45122300
C	6.14370500	-2.03676900	0.62075100
H	6.64849700	-1.06435100	0.65124800
H	6.88338000	-2.73931400	0.20308800
C	4.98252000	-1.95370500	-0.37097300
H	4.11697000	-1.23647000	0.18122900
C	5.37697700	-1.23234400	-1.65879800
H	6.22417600	-1.78561600	-2.09798000
H	5.77481300	-0.24568300	-1.39680400
C	4.28775700	-1.06672700	-2.72606400
H	3.39834900	-0.61825700	-2.27290900
H	3.98080800	-2.04846900	-3.10153500
C	4.75727100	-0.20345800	-3.90138700
H	5.63833600	-0.63628800	-4.38700400
H	3.97521500	-0.10701100	-4.65978300
H	5.02292800	0.80396500	-3.56760100
C	4.22093300	-3.25612900	-0.56730500
H	3.31886400	-3.10569600	-1.16078100
H	4.85703300	-3.98445300	-1.09065200
H	3.92065100	-3.69624800	0.38457300
C	4.07986500	3.77102800	-0.69927700
C	3.18129000	2.73348600	-0.51328900
C	3.54767100	1.56423700	0.20856500
C	4.86971000	1.51003500	0.72605900
C	5.75853800	2.55753100	0.53427300
C	5.37919800	3.69716100	-0.18130500
H	3.77280800	4.64596000	-1.26267600
H	2.18870400	2.80053400	-0.94257200
H	5.16734500	0.65143500	1.31363500
H	6.75614400	2.49370000	0.95671800
H	6.07829300	4.51151100	-0.33103900
C	2.62756900	0.48296000	0.39807100
C	1.17324700	0.63933700	0.44095200
C	0.29204700	-0.29926100	-0.14601300
C	0.57658300	1.71872700	1.13152600
C	-1.09449300	-0.15333900	-0.04842000
C	-0.79050400	1.88056400	1.22700900
H	1.22940300	2.43862500	1.61000200
C	-1.64211200	0.93296800	0.63749200
H	-1.73121700	-0.88866400	-0.52606300
H	-1.22583300	2.71416100	1.76351600
O	3.01500500	-0.74642700	0.78635900
O	0.82373100	-1.32979300	-0.86546300
H	0.11022300	-1.86319600	-1.23088400

O	-2.97554900	1.15287500	0.78357200
C	-3.90618400	0.22432600	0.22054800
H	-3.75825200	0.16841500	-0.86565200
H	-3.73057200	-0.77333000	0.64301800
C	-5.31037200	0.70706500	0.54667300
H	-5.43703300	1.71714500	0.14311000
H	-5.41050300	0.78618200	1.63424600
C	-6.39081500	-0.22391100	-0.01574400
H	-6.27218800	-0.30756600	-1.10377800
H	-6.24826400	-1.23544500	0.38582500
C	-7.81614500	0.24504500	0.29872100
H	-7.93759200	0.32747000	1.38619700
H	-7.95982500	1.25691700	-0.10085500
C	-8.90066900	-0.68050400	-0.26386600
H	-8.77692400	-0.76301100	-1.35153900
H	-8.75525100	-1.69259300	0.13571300
C	-10.32744300	-0.21527800	0.04746100
H	-10.45312100	-0.13333400	1.13500500
H	-10.47403800	0.79697800	-0.35143800
C	-11.41235200	-1.14013200	-0.51618800
H	-11.28735000	-1.22197100	-1.60285900
H	-11.26677700	-2.15138400	-0.11702500
C	-12.83459200	-0.66763600	-0.20120600
H	-13.02230600	0.32710400	-0.61774500
H	-13.58318500	-1.34780700	-0.61691900
H	-13.00228500	-0.60977400	0.87894500

TSHBp

C	2.73379900	-1.63242300	0.02954000
C	2.32121000	-0.33217500	-0.37951800
C	3.34434200	0.57148600	-0.76939500
C	4.68344600	0.22228100	-0.71955300
C	5.06456200	-1.05652700	-0.30166100
C	4.08028600	-1.97454800	0.06558700
C	0.92389600	0.03565700	-0.43965900
C	0.41983800	1.39707300	-0.46648300
C	1.01624000	2.43342300	0.28949500
C	0.51211300	3.72620100	0.25215200
C	-0.60086700	4.03496000	-0.53541300
C	-1.20538000	3.02345200	-1.28488700
C	-0.71154600	1.72582500	-1.24911000
O	0.08692000	-0.99478700	-0.65502600
C	-2.24574600	-1.04474600	0.51696600
C	-3.32587700	-0.83247800	-0.54676200

C	−3.20828500	−1.66467200	−1.83123900
C	−4.28969900	−1.30911700	−2.85643600
C	−2.32886500	0.01634200	1.61807400
C	−1.25830600	0.00151700	2.72062600
C	−1.76163100	0.62346100	4.02786600
C	−2.11896500	−2.48362000	0.99772100
H	−4.22661200	−0.25730700	−3.15317700
H	−4.19199700	−1.91470000	−3.76144500
H	−5.29259700	−1.47739100	−2.45089800
H	−2.21659800	−1.51382000	−2.27089100
H	−3.27814200	−2.73094200	−1.59479900
H	−3.35137300	0.23068400	−0.81027400
H	−4.29850200	−1.04334700	−0.07345800
H	−2.01219500	−3.17636100	0.16215200
H	−1.25720200	−2.62412000	1.65000900
H	−3.01970400	−2.76920700	1.55905900
H	−2.34752900	1.00540400	1.14865900
H	−3.31811400	−0.10464100	2.09034200
H	−0.37274500	0.54179700	2.37335300
H	−0.93278100	−1.02229000	2.92287700
H	−2.09220000	1.65613600	3.87753600
H	−2.60913800	0.05987500	4.43084400
H	−0.97705800	0.63503900	4.78946700
H	−1.18085000	−0.86497700	−0.05907400
H	−1.17102800	0.95507300	−1.85489700
H	−2.06210100	3.25205900	−1.91002700
H	−0.98990600	5.04614300	−0.56297600
H	0.98247800	4.49908400	0.85053500
H	1.86205600	2.20688300	0.92729600
O	1.77517100	−2.54395800	0.43431200
H	4.36492900	−2.96989500	0.39320400
H	6.11096900	−1.33587500	−0.26843100
H	5.43596400	0.94119800	−1.02399300
H	3.06505500	1.55103900	−1.13410800
H	2.21279800	−3.35381500	0.69135200

TSDHBp

C	−1.22089300	1.78370500	−1.04719600
C	0.02942200	1.50415700	−0.43377000
C	0.72746700	2.59905400	0.14543500
C	0.20463000	3.88114100	0.10901800
C	−1.03044000	4.13548800	−0.50058600
C	−1.73138500	3.07317200	−1.07896400
C	0.56031500	0.17383500	−0.39547300

O	-0. 18666700	-0. 91836900	-0. 64068900
C	1. 98902600	-0. 13963300	-0. 34226600
C	2. 50031700	-1. 20630600	0. 43923700
C	3. 86180600	-1. 51012000	0. 43022100
C	4. 74742300	-0. 76601100	-0. 34940000
C	4. 27461400	0. 29516800	-1. 12627200
C	2. 92094800	0. 58047700	-1. 12026200
O	1. 64140800	-1. 89249500	1. 24539200
C	-2. 49114300	-1. 25560800	0. 48109800
C	-2. 17655400	-2. 66612900	0. 95823200
C	-3. 55672100	-1. 17747400	-0. 61366600
C	-3. 26967100	-1. 94040200	-1. 91471300
C	-4. 36072700	-1. 72632700	-2. 96845700
C	-2. 73069800	-0. 23396300	1. 59126200
C	-1. 70022300	-0. 18135200	2. 72651700
C	-1. 97957000	0. 96225500	3. 70694800
H	-4. 45465600	-0. 66849000	-3. 23391100
H	-4. 13947800	-2. 27898000	-3. 88557400
H	-5. 33719600	-2. 06314800	-2. 60538400
H	-2. 30090000	-1. 62150800	-2. 31267100
H	-3. 17755200	-3. 01124100	-1. 70788500
H	-3. 73979200	-0. 12296500	-0. 85017200
H	-4. 50020500	-1. 55005800	-0. 18252700
H	-1. 96385800	-3. 33613100	0. 12409400
H	-1. 31179100	-2. 68110000	1. 62199100
H	-3. 03702100	-3. 07320300	1. 50821900
H	-2. 81451900	0. 76003500	1. 13801700
H	-3. 72320900	-0. 44769200	2. 02233200
H	-0. 69757800	-0. 06491400	2. 30353900
H	-1. 69656200	-1. 13017900	3. 27296600
H	-1. 94033600	1. 93184600	3. 20175300
H	-2. 97083300	0. 86383300	4. 16195900
H	-1. 24405200	0. 97912400	4. 51603800
H	-1. 42084500	-0. 93381600	-0. 07336200
H	-1. 75833800	0. 97921900	-1. 53211000
H	-2. 68068100	3. 25823400	-1. 57116200
H	-1. 43463400	5. 14059500	-0. 52617600
H	0. 75713300	4. 69416500	0. 56817100
H	1. 67372100	2. 42009000	0. 64193600
H	4. 23401200	-2. 32344700	1. 04945600
O	6. 08665700	-1. 03081700	-0. 38845200
H	4. 97006800	0. 86046000	-1. 73343000
H	2. 54818500	1. 38431800	-1. 74316200
H	2. 13040700	-2. 55619200	1. 74297300

H	6.29166200	-1.78616500	0.17242400

TSMBp

C	3.98105400	-1.63192500	3.05376100
H	5.01709000	-1.82319700	2.75598100
H	3.92838500	-0.60269000	3.42267500
H	3.74897500	-2.29537500	3.89111600
C	3.01475100	-1.85464300	1.88617100
H	3.06533800	-2.90303900	1.57592200
H	1.98575200	-1.68325500	2.21848900
C	3.32079000	-0.93890300	0.69250600
H	3.36269100	0.10005500	1.03811200
H	4.33311100	-1.16510400	0.31999800
C	2.37079300	-1.02287100	-0.50351400
H	1.22397200	-0.85414400	-0.03238300
C	2.59876600	0.10701600	-1.50688600
H	3.62446400	-0.00694300	-1.89540800
H	2.58939000	1.06059600	-0.96851400
C	1.62852900	0.19461900	-2.69144000
H	0.60198700	0.24654300	-2.31388400
H	1.69295200	-0.71325000	-3.29957800
C	1.90501900	1.41259600	-3.57880400
H	2.91965100	1.38285800	-3.98928700
H	1.20834000	1.45536100	-4.42035900
H	1.80144400	2.34351300	-3.01371800
C	2.25914000	-2.41346700	-1.11213100
H	1.51934500	-2.45244400	-1.91186500
H	3.22887500	-2.70825800	-1.53724200
H	1.98226600	-3.16038900	-0.36681300
C	-0.84007500	3.74010100	0.04124100
C	-1.25068400	2.42267200	-0.08947600
C	-0.48654500	1.35948200	0.45695600
C	0.70829900	1.70131100	1.13745800
C	1.10832300	3.02475600	1.26537700
C	0.34357300	4.05710400	0.71758400
H	-1.44069600	4.53055400	-0.39601400
H	-2.15917900	2.19561900	-0.63409000
H	1.29131100	0.91721700	1.60224200
H	2.01782500	3.25675000	1.80982900
H	0.66018500	5.08872300	0.81713400
C	-0.90575800	-0.01332800	0.31879800
C	-2.30497600	-0.44654000	0.28715600
C	-2.74442100	-1.57069100	-0.47137600
C	-3.25214200	0.24830400	1.07544400

C	-4.09479100	-1.92158200	-0.41652500
C	-4.58696600	-0.12210200	1.10674900
H	-2.91453000	1.08169600	1.67897500
C	-5.01617100	-1.21893600	0.35802200
H	-4.43563300	-2.76452300	-1.00923700
H	-5.28702000	0.43102700	1.72297000
H	-6.05612200	-1.52481700	0.37571000
O	-0.04156900	-1.03861500	0.43674900
C	-1.82606900	-2.35526500	-1.37627600
H	-1.18995800	-1.69053200	-1.96588100
H	-1.16063700	-3.00923200	-0.80908000
H	-2.40965700	-2.97065200	-2.06456300

TSBp

C	-1.30530900	0.44965200	4.00913600
C	-1.21884500	-0.55827300	2.85877500
C	-2.21776800	-0.24773400	1.73671000
C	-2.21752400	-1.18139300	0.53229800
C	-2.21297600	-2.66488900	0.85743700
C	-3.21823700	-0.78501800	-0.54923800
C	-2.93399100	-1.27142900	-1.97954700
C	-4.05918900	-0.90770100	-2.95235900
H	-4.17735100	0.17734000	-3.03734400
H	-3.85597300	-1.29726200	-3.95337100
H	-5.01914200	-1.31906000	-2.62426600
H	-1.98862100	-0.85080000	-2.33035600
H	-2.79415900	-2.35738400	-1.97998900
H	-3.33388600	0.30410500	-0.55316800
H	-4.19745700	-1.18930700	-0.24276900
H	-2.21887500	-3.27849100	-0.04490600
H	-1.34748600	-2.95614800	1.45420900
H	-3.11369400	-2.91532900	1.43685700
H	-2.06788800	0.78024700	1.39066000
H	-3.23800900	-0.26986600	2.16012100
H	-0.20306300	-0.55831200	2.44979700
H	-1.39554000	-1.56464400	3.25096200
H	-1.10670100	1.46658600	3.65834200
H	-2.29885900	0.44375200	4.46906200
H	-0.57667900	0.21928000	4.79099600
H	-1.07735600	-1.02957000	0.02764000
O	0.12484800	-1.15210300	-0.57190100
C	0.97758400	-0.11978200	-0.38266100
C	0.48193700	1.24490600	-0.37706800
C	1.11613300	2.26953800	0.36458900

C	0.62218300	3.56703700	0.36109600
C	-0.51952900	3.89330100	-0.37622500
C	-1.16392500	2.89380800	-1.10800800
C	-0.67956800	1.59199700	-1.10481500
H	-1.17291900	0.83369500	-1.69880200
H	-2.04421300	3.13478400	-1.69453000
H	-0.89973900	4.90821600	-0.37898700
H	1.12354300	4.32931100	0.94780200
H	1.98478200	2.03020700	0.96556800
C	2.36855700	-0.50616900	-0.36235200
C	3.41773200	0.39342700	-0.68368500
C	4.74040200	-0.02042900	-0.67140900
C	5.07342200	-1.34274100	-0.35740700
C	4.05237900	-2.25182600	-0.06853600
C	2.72491500	-1.84999500	-0.07377700
H	1.93925900	-2.55899400	0.15045400
H	4.29679100	-3.28229100	0.16642900
H	6.10983400	-1.65983100	-0.34959300
H	5.52175600	0.68841600	-0.92383800
H	3.18255000	1.41045400	-0.96948100

TSAFBp

C	2.06638100	-2.11065000	0.19483200
C	1.77097600	-0.75128000	-0.18297000
C	2.87092700	0.08811200	-0.50929900
C	4.17017900	-0.35023500	-0.39561200
C	4.46042700	-1.65826200	0.01193200
C	3.40835700	-2.51430300	0.29182500
C	0.42753500	-0.22871800	-0.26486800
C	0.11848700	1.18827300	-0.12896900
C	0.75434100	1.98831400	0.85121100
C	0.46619600	3.33640600	1.00306300
C	-0.48080100	3.95237200	0.18065400
C	-1.12520000	3.20039700	-0.80084800
C	-0.81940600	1.85820300	-0.93860400
O	-0.52341600	-1.11517900	-0.61668000
C	-2.88615700	-0.95952100	0.46533300
C	-3.85286300	-0.49588600	-0.62519200
C	-3.76786500	-1.21717600	-1.97725200
C	-4.70577400	-0.60262600	-3.02074400
C	-2.86239500	-0.01108200	1.66304700
C	-1.93402200	-0.37778600	2.82826900
C	-1.86217300	0.73057600	3.88334500
C	-3.00250300	-2.43687300	0.81646200

H	-4.44307000	0.44061800	-3.21985100
H	-4.65160800	-1.14455700	-3.96880400
H	-5.74773200	-0.62564300	-2.68450900
H	-2.73684600	-1.17634900	-2.33989000
H	-4.01809000	-2.27608700	-1.85323800
H	-3.71116000	0.57744400	-0.78647000
H	-4.87322800	-0.60809900	-0.22253100
H	-2.92095200	-3.07056800	-0.06844500
H	-2.23875500	-2.74945600	1.53008900
H	-3.98299900	-2.63321700	1.27285900
H	-2.61660100	0.99585300	1.30825000
H	-3.89547400	0.05701400	2.04254100
H	-0.92830000	-0.58161000	2.44525000
H	-2.27672700	-1.30308800	3.30228200
H	-1.48467900	1.66127500	3.44995900
H	-2.84894500	0.93808000	4.31011000
H	-1.19814900	0.45118900	4.70584400
H	-1.75726300	-0.89441100	-0.06093500
F	-1.43310600	1.17975300	-1.94232400
H	-1.84631400	3.64381700	-1.47699200
H	-0.71238500	5.00439400	0.29517200
H	0.97283700	3.90824900	1.77189900
H	1.47418500	1.51592900	1.50847600
N	1.07059100	-2.99731500	0.54661600
H	3.62157300	-3.53281900	0.60056300
H	5.48580500	-1.99387700	0.09337200
Cl	5.49794500	0.74159700	-0.78643200
H	2.67693900	1.09397100	-0.85421700
H	1.33622700	-3.96546900	0.63660100
H	0.15991300	-2.82440000	0.14631500

TSABp

C	4.32263400	-1.41493700	-2.89913100
H	5.31264600	-1.66217500	-2.50238600
H	3.79721900	-2.35619900	-3.08841900
H	4.46621400	-0.91684500	-3.86172000
C	3.53791100	-0.52801700	-1.92727400
H	4.07366000	0.41734900	-1.79563400
H	2.56391100	-0.28132900	-2.36169100
C	3.32011000	-1.20462800	-0.56749300
H	2.85758100	-2.18501800	-0.73258000
H	4.30050200	-1.41225000	-0.10815800
C	2.48358300	-0.44038600	0.46139600
H	1.38254300	-0.32119300	-0.14295300

C	2.15116100	-1.30052100	1.68216500
H	3.10354200	-1.52777300	2.18913700
H	1.76364100	-2.26633300	1.33806900
C	1.17411700	-0.70963200	2.70626000
H	0.24414400	-0.43207200	2.20005700
H	1.58585600	0.21204200	3.12912800
C	0.86190600	-1.68827100	3.84259300
H	1.77048800	-1.97807700	4.38076400
H	0.17448300	-1.24444100	4.56766400
H	0.39495500	-2.60055000	3.45934800
C	2.99010000	0.95176800	0.79409200
H	2.28109900	1.51485000	1.40137600
H	3.92620300	0.86812700	1.36564000
H	3.20018100	1.54121900	-0.09821100
C	-1.35308900	3.66730500	0.91836600
C	-1.51790300	2.30903700	0.68922600
C	-0.67155800	1.61162900	-0.20606100
C	0.34972400	2.35024600	-0.84782200
C	0.50261400	3.71163100	-0.61810300
C	-0.34371700	4.38320400	0.26621900
H	-2.00891300	4.17336900	1.61876600
H	-2.29139500	1.76567900	1.21874900
H	0.99836900	1.84341800	-1.55131900
H	1.28248400	4.25717400	-1.13919300
H	-0.21980900	5.44477900	0.44639300
C	-0.81984800	0.18975100	-0.44984400
C	-2.08990000	-0.49648800	-0.54716100
C	-2.23456100	-1.91188200	-0.32365900
C	-3.26240800	0.22405500	-0.90179700
C	-3.51387200	-2.48847300	-0.38913900
C	-4.50928400	-0.36597500	-0.95811000
H	-3.16127400	1.27536900	-1.13776400
C	-4.63838400	-1.73762100	-0.69164400
H	-3.61052500	-3.55254600	-0.19493800
H	-5.37799400	0.22573500	-1.22204000
H	-5.60955900	-2.21689300	-0.73810700
O	0.25549600	-0.53389000	-0.85384800
N	-1.15938000	-2.70109000	0.04010200
H	-0.25855800	-2.37962100	-0.28405500
H	-1.30600200	-3.69781200	-0.00432900

TSHMBp

C	-0.70336500	3.70511600	0.52326500
C	0.01044100	2.52104900	0.42683800

C	-0.47548000	1.43645000	-0.34329500
C	-1.71541000	1.60952100	-1.00542700
C	-2.41984000	2.80174500	-0.90819000
C	-1.92522300	3.85983600	-0.14125300
H	-0.31202500	4.51593600	1.12875200
H	0.94503600	2.41281200	0.96359400
H	-2.09215500	0.80839700	-1.62855500
H	-3.35739500	2.91361000	-1.44293800
H	-2.47921500	4.78812900	-0.06288600
C	0.24271500	0.19113100	-0.43048500
C	1.67806200	0.00027700	-0.34012000
C	2.64647200	0.92185500	-0.83961200
C	2.18761700	-1.21274100	0.17904900
C	4.00325600	0.66226800	-0.73242200
C	3.54389400	-1.49021200	0.27193500
H	1.47264000	-1.94600200	0.52704400
C	4.46519500	-0.53780300	-0.17732200
H	4.73298500	1.37268600	-1.10776000
H	3.86763900	-2.43069100	0.69577400
O	-0.40319900	-0.97156100	-0.67985600
O	2.19796400	2.05315500	-1.45458400
H	2.94823800	2.56128800	-1.78121100
O	5.81927900	-0.67906100	-0.13226900
C	6.36045900	-1.88355800	0.39685300
H	6.04276800	-2.75315900	-0.18777000
H	7.44181600	-1.77743400	0.32888600
H	6.07420800	-2.02214800	1.44462600
C	-4.65082800	-1.91363800	-2.83941300
H	-5.59671400	-2.29741500	-2.44353100
H	-4.78978100	-0.84872100	-3.05099600
H	-4.46063500	-2.41783800	-3.79074900
C	-3.50130800	-2.13940000	-1.85238000
H	-3.36611200	-3.21532000	-1.70267000
H	-2.56448200	-1.77022500	-2.28226500
C	-3.74571000	-1.44742400	-0.50390400
H	-3.97999600	-0.39180800	-0.67984700
H	-4.64886200	-1.87738300	-0.04082800
C	-2.62069300	-1.52971400	0.52840700
H	-1.60063600	-1.11386400	-0.06403400
C	-2.85250700	-0.58301400	1.70509200
H	-3.81225900	-0.86650300	2.16846600
H	-3.00164200	0.42829200	1.31161700
C	-1.77291600	-0.54044400	2.79447300
H	-0.79908600	-0.34238700	2.33450800

H	-1.69403200	-1.51618800	3.28495000
C	-2.06134500	0.53055700	3.85117200
H	-3.02330800	0.35374300	4.34356300
H	-1.28948400	0.54067000	4.62565800
H	-2.09545500	1.52674200	3.40081400
C	-2.21329900	-2.94663800	0.90579600
H	-1.34431600	-2.95718300	1.56499200
H	-3.03917000	-3.44297800	1.43499100
H	-1.97203800	-3.54663400	0.02714500

TSNBp

C	0.14424300	-3.87426800	0.77566500
C	-0.64279900	-2.88085200	1.32561300
C	-0.28911900	-1.53258800	1.18308900
C	0.84823600	-1.13519200	0.41801600
C	1.64774800	-2.19474900	-0.09671500
C	1.31093600	-3.52054700	0.07332300
C	1.18360800	0.21250900	0.03360600
O	0.31262700	1.16533700	-0.21295000
N	-1.04231200	-0.58523900	1.98351900
O	-0.51228800	0.48715200	2.28038300
C	2.56832500	0.67690200	-0.11612400
C	3.60743700	0.16172600	0.68623700
C	4.90486300	0.63480800	0.54718600
C	5.19445400	1.64576900	-0.37326200
C	4.16979000	2.18372200	-1.15315800
C	2.86941800	1.71283400	-1.02540100
O	-2.17889000	-0.91434500	2.35701300
C	-2.15017800	0.96580800	-1.08616400
C	-1.85568000	1.89139000	-2.25897100
C	-3.02672400	1.58425300	0.00640500
C	-2.53302000	2.88779000	0.64894600
C	-3.47606300	3.37231300	1.75444200
C	-2.61567600	-0.43783300	-1.47580100
C	-1.76914200	-1.20349800	-2.50031300
C	-2.30588300	-2.61600600	-2.75412800
H	-4.48315400	3.56100100	1.36737300
H	-3.55727400	2.62934800	2.55279800
H	-3.11265500	4.30179800	2.20104600
H	-2.43604100	3.67157700	-0.10982100
H	-1.53666500	2.72229900	1.06736300
H	-3.19338300	0.84140800	0.79222100
H	-4.01349600	1.76408700	-0.45272500
H	-1.04798700	0.84886600	-0.57077300

H	-3.63785400	-0.32963700	-1.87399900
H	-2.72140100	-1.03945500	-0.56720100
H	-0.73429700	-1.26706500	-2.14708400
H	-1.74004600	-0.65692000	-3.44816900
H	-3.33217300	-2.58709400	-3.13400900
H	-1.69448600	-3.14540600	-3.48981400
H	-2.30828400	-3.20853100	-1.83460600
H	-1.14956400	1.45039600	-2.96392800
H	-2.78784200	2.09601100	-2.80342900
H	-1.44821200	2.84666400	-1.92659800
H	5.69286500	0.22701200	1.17031300
H	3.38477900	-0.59507500	1.42780200
H	2.07481200	2.12672800	-1.63209000
H	4.38726700	2.97158200	-1.86556000
H	6.20828000	2.01590300	-0.47383200
H	2.51418800	-1.93788900	-0.69235600
H	-1.52172900	-3.11635600	1.90965500
H	1.93860800	-4.29064200	-0.36031600
H	-0.12831100	-4.91481800	0.90259300
